# Low-Dose and Scatter-Free Cone-Beam CT Imaging Using a Stationary Beam Blocker in a Single Scan: Phantom Studies

**DOI:** 10.1155/2013/637614

**Published:** 2013-11-20

**Authors:** Xue Dong, Michael Petrongolo, Tianye Niu, Lei Zhu

**Affiliations:** Nuclear & Radiological Engineering and Medical Physics Programs, The George W. Woodruff School of Mechanical Engineering, Georgia Institute of Technology, Atlanta, GA 30332, USA

## Abstract

Excessive imaging dose from repeated scans and poor image quality mainly due to scatter contamination are the two bottlenecks of cone-beam CT (CBCT) imaging. Compressed sensing (CS) reconstruction algorithms show promises in recovering faithful signals from low-dose projection data but do not serve well the needs of accurate CBCT imaging if effective scatter correction is not in place. Scatter can be accurately measured and removed using measurement-based methods. However, these approaches are considered unpractical in the conventional FDK reconstruction, due to the inevitable primary loss for scatter measurement. We combine measurement-based scatter correction and CS-based iterative reconstruction to generate scatter-free images from low-dose projections. We distribute blocked areas on the detector where primary signals are considered redundant in a full scan. Scatter distribution is estimated by interpolating/extrapolating measured scatter samples inside blocked areas. CS-based iterative reconstruction is finally carried out on the undersampled data to obtain scatter-free and low-dose CBCT images. With only 25% of conventional full-scan dose, our method reduces the average CT number error from 250 HU to 24 HU and increases the contrast by a factor of 2.1 on Catphan 600 phantom. On an anthropomorphic head phantom, the average CT number error is reduced from 224 HU to 10 HU in the central uniform area.

## 1. Introduction

Onboard cone-beam CT (CBCT) is being increasingly implemented on radiation therapy machines for accurate patient positioning and tumor targeting in image-guided radiation therapy (IGRT). The use of CBCT increases patient setup accuracy and also opens possibilities of CBCT-based accurate tumor delineation and therapeutic dose calculation. Nevertheless, the wide application of CBCT in IGRT is limited by excessive imaging dose and poor image quality.

The repeated CBCT scans during the treatment procedure produce high dose to healthy organs. It has been reported that the dose delivered from a CBCT system could be as high as 5~10 cGy per scan and 100~300 cGy per treatment course [1–6]. Although radiotherapy patients are being exposed to higher radiation doses for cancer treatment, the additional CBCT dose leads to skin burns cataracts, and increased risks of radiation-induced cancer or genetic defects [1]. Moreover, the CBCT dose is particularly risky for radiation-sensitive groups [5]. For example, CBCT-guided radiation therapy is essentially prohibitive for pediatric patients, resulting in suboptimal treatment outcomes. Patient dose can be lowered by optimizing both hardware and software designs of the CT systems. Existing approaches include optimization of data acquisition protocols (e.g., automatic exposure control), improvement of detector quantum efficiency, region-of-interest (ROI) reconstruction [7] from reduced projections, and noise suppression with degraded spatial resolution. However, after continuous development of CT systems for decades, further dose reduction from these techniques is limited or costly. Decreasing the total number of incident photons of each projection ray (i.e., mAs) and reducing the number of X-ray projections also lower the patient dose but with degraded image qualities in the conventional filtered back projection (FBP) reconstruction [8]. The recent advances in compressed sensing (CS) enable accurate CT image recovery from undersampled data [9]. Compared to the analytical algorithms, total variation- (TV-) based CS methods [10, 11] have demonstrated significant improvements in both fan-beam and cone-beam CT reconstruction especially when projection data are undersampled with sparse views [11–13] or with missing data in a single view [11]. These reconstruction algorithms minimize the TV of the CT image constrained by the data fidelity and image nonnegativity, which show promises in reducing CT dose without significantly degrading image qualities.

Besides excessive patient dose, CBCT images are also subject to severe contamination from scatter radiation. Scatter signals induce large image artifacts and CT number nonlinearity, which limit the applications of CBCT. For a middle-size human torso, the average scatter-to-primary ratio (SPR) is around 2~3, which leads to CT number errors up to 350 HU [14–17]. Extensive studies have been conducted on scatter correction techniques. These published techniques can be divided into two major categories, based on whether scatter signals are directly measured or not. Nonmeasurement-based methods either prevent scattered radiation from reaching the detector (e.g., using an antiscatter grid [18, 19], limiting the field of view (FOV), and increasing the air gap between the object and the detector [20]) or predict the scatter distribution (using, e.g., analytical modeling [21], modulation methods [22–25], and Monte Carlo (MC) simulation [26, 27]). These methods improve the image quality to a certain extent, but their performances are limited in clinical applications [28]. An anti-scatter grid results in primary signal loss, thus, increasing image noise and degrades image qualities [18, 19]. The air-gap between the object and the detector is limited by the size of operation room [20]. Monte Carlo simulation generates accurate scatter signals but is computationally intense [26, 27]. On the other hand, methods of direct scatter measurement conveniently obtain accurate scatter estimates with negligible computational cost [17]. In the measurement-based method, a beam blocker is typically inserted between the X-ray source and the object, and scatter signals are estimated inside the detector shadows of the beam blocker [15, 29–31], where primary signals are fully attenuated. The scatter distribution of the whole field is then obtained via interpolation/extrapolation on the scatter samples inside the shadows, since scatter distributions have dominant low-frequency components [15, 32, 33]. The method achieves accurate scatter estimation without prior knowledge of X-ray source, object, imaging geometry, and is easy to implement. Nonetheless, primary signal loss is inevitable due to the insertion of the beam blocker. As a result, severe image artifacts appear in the conventional [34] reconstruction if the missing primary signals are not compensated for [17]. Two projections per view, one with the blocker and the other without [14], or moving blockers during the scan [35], are designed to compensate for the primary loss. These hardware modifications complicate the data acquisition and increase scan time and patient dose. Recently, we developed a “crossing-finger”-shape beam blocker, which makes use of the data redundancy condition in a 360-deg full-fan CT scan. This method achieves accurate scatter estimation and reconstruction within one single scan and thus is considered clinically more attractive. Though demonstrated promise, the “crossing-finger”-shape blocker is of complex structure, and the insertion of beam blocker complicates the FDK reconstruction algorithm.

For years research has been developed independently on dose reduction and scatter correction. Nevertheless, little effort has been devoted to exploit the full potential of image improvement from a combination of the above two schemes. Scatter measurement accurately corrects for scatter but leads to primary loss, which makes most of the measurement-based correction methods unpractical. CS-based iterative algorithm lowers imaging doses and obtains accurate reconstruction even on the insufficient data from sparse views or a reduced number of detector pixels. Considering the complimentary capabilities of these two approaches, in this work, we propose to use an improved stationary beam blocker in the CBCT system for simultaneous dose reduction and scatter measurement and an iterative algorithm for accurate reconstruction on the projections with missing data in a single scan. The new method explores the strengths of measurement-based scatter correction and iterative reconstruction while eliminates their shortcomings and obtains low-dose and scatter-free CBCT images.

In the new method, the lead strips of the blocker are placed in the longitudinal direction and located asymmetrically with respect to the central longitudinal line of the detector. If one ray is blocked by the strip, its conjugate is still measured after around 180-deg rotation even if it is in the off-plane. We insert the beam blocker between the X-ray source and the object, where scatter distribution is obtained by interpolation/extrapolation on the scatter samples inside the strip shadow. The insertion of blocker also reduces patient dose since X-ray primary signals are attenuated [36]. We further reduce the patient dose by decreasing the projection number. Our recently developed CS-based iterative reconstruction, accelerated barrier optimization for compressed sensing (ABOCS) [8], is carried out on the blocked data to obtain scatter-free and low-dose CBCT images. We evaluate the performance of the method on the Catphan 600 phantom and an anthropomorphic head phantom.

## 2. Method

### 2.1. Blocker Design

In a circular cone-beam CT scan, one projection ray can be specified by (*θ*, *φ*, *α*), where *θ* and *φ* are the angles of the ray in the transverse and axial directions, respectively, and *α* is the projection angle of the source. It can be easily verified that no projection rays are redundant in such geometry except those in the midplane (i.e., *φ* = 0). Nevertheless, if we employ a commonly used approximation of small cone angle (i.e., *φ* ≈ 0 for the whole projection), the redundant rays have the following relationships:

(1) $$\begin{array}{c} \theta_{1}={-\theta }_{2},\\ \left|\alpha_{1}-\alpha_{2}\right|+2\left|\theta_{1}\right|=\pi , \end{array}$$

with a full rotation and full object coverage; half of the CBCT projection data are considered to be redundant. Under the small-cone-angle approximation, each projection ray in a CBCT full scan has a corresponding redundant ray measured from the opposing direction. The two lines are referred to as a conjugate ray pair and this condition is referred to as the data redundancy. We can therefore block some of these redundant rays for other purposes (e.g., scatter measurement) while still maintaining an accurate reconstruction [37]. No hardware compensation for the missing primary data is necessary and the data acquisition is complete with one single-scan.

Guided by this principle, we place lead strips in the longitudinal direction, which is perpendicular to the rotation plane, to block only redundant rays for scatter measurement. One ray blocked by the strip is measured through its conjugate after around 180-deg rotation. The beam blocker is designed to block less than 50% of full illuminated field and are placed asymmetrically with respect to the central longitudinal line of the detector, such that at least one ray from its conjugate ray pair can be measured on the detector. Note that, the central longitudinal line of the detector is always left unblocked to avoid the missing rays passing through the object center.

**Figure 1:**
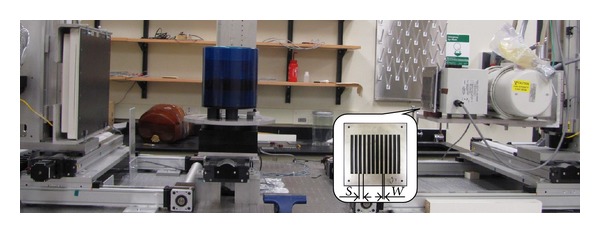
The CBCT tabletop system and Catphan 600 phantom. The designed blocker is mounted in front of the collimator and shown in an enlarged insert. The lead blocker is sandwiched between two layers of thin steel, each with a thickness of 0.2 mm, to improve the mechanical strength.

Figure 1 shows the geometry of the proposed method and our experimental setup. The designed blocker is placed between the X-ray source and the object. The lead strips are placed along the longitudinal direction and uniformly distributed in the lateral direction. The strips have a thickness of around 3 mm and attenuate more than 99.99% of incident X-ray photons. Only scatter samples are measured inside the shadows on the detector. Besides the strip placement, two more parameters are needed in the blocker design: sampling period (*S*) and strip width (*W*). To guarantee the measurement of at least one ray from its conjugate pair, we choose a relatively large sampling period (*S* ≈ 52 mm on the detector) based on the observed maximum spatial frequency of scatter signals in our previous studies [15, 17, 38] as well as in the literature [39]. The strip width cannot be too small since the penumbra effects on the strips limit scatter measurement accuracy [38]. Moreover, wider blocker contributes more to the dose reduction. Based on our previous study [16], *W* is chosen as about 17 mm on the detector, which blocks 33% of the illuminated area.

### 2.2. Scatter Estimation and Correction

The tabletop system geometry is shown in Figure 1. As shown in our previous studies [16, 17, 24] and the literature [39], the insertion of the beam blocker does not greatly perturb the spatial frequency spectrum of scatter in cone-beam projections and scatter is still predominantly low-frequency. The whole field scatter distribution is therefore accurately estimated using interpolation/extrapolation on the measured samples. To avoid the penumbra effect of the strips, only the measured data inside the central two-third of the strip shadows are used in the scatter estimation. Since the lead strips cover the whole blocker in the longitudinal direction, a one-dimensional (1D) cubic interpolation is carried out on each lateral line to estimate the scatter distribution over the whole detector area. The estimated scatter is then subtracted from the raw projection to generate the scatter-corrected CBCT projection.

### 2.3. Reconstruction on the Incomplete Data

These corrected CBCT projections are incomplete due to the insertion of the blocker and the angular undersampling. Severe artifacts therefore appear in the conventional FDK reconstruction. To improve the image quality, we first compensate for the missing primary in the blocked area using their conjugate rays. As described by (1), the two detector points corresponding to the conjugate ray pair are symmetric with respect to the detector central longitudinal line, and their projection angle has a difference of *π* − 2 | *θ*|. Due to the discretization of the data acquisition in both spatial and angular directions, the missing primary is compensated for using its conjugate point by interpolating on the scatter-corrected sinogram.

An in-house CS-based iterative reconstruction is applied to further improve the image quality. The algorithm is referred to as the accelerated barrier optimization for compressed sensing (ABOCS) reconstruction algorithm, which minimizes the image TV term with data fidelity and nonnegativity constraints [8]. ABOCS formulates the TV minimization constrained by the data fidelity into a form similar to that of the conventional TV regularization but with an automatically adjusted penalty weight. The automatic penalty weight is controlled by the data fidelity tolerance, which is estimated from the raw projections according to the Poisson statistics and the data error in the current iteration. Consistent reconstruction performances are achieved using the same algorithm parameters on scans with different noise levels and/or on different objects. The problem is then solved efficiently by gradient projection with an adaptive Barzilai-Borwein step-size selection scheme. Readers are referred to [8] for more details. Note that, image noise increases significantly after scatter correction [14]. An additional penalized weighted least-squares (PWLS) algorithm [14] is performed to reduce the noise in the reconstructed images.

### 2.4. Evaluation

The performance of the proposed method is evaluated on the Catphan 600 phantom with a diameter of 200 mm (The Phantom Laboratory, Salem, NY) and an anthropomorphic head phantom on our CBCT table-top system. The geometry of this system exactly matches that of a Varian On-board Imager (OBI) CBCT system on the TrueBeam radiation therapy machine. A detailed system configuration is described in [16]. The lead sheet of the designed blocker is first shaped using a waterjet cutting system. To improve the mechanical strength of the blocker, the lead is then sandwiched between two layers of thin steel (~0.2 mm) using J-B WELD epoxy adhesive (http://www.grainger.com/).

CBCT images are compared with and without the proposed method. A total of 655 projections are acquired for the conventional FDK reconstruction. Few-view projection data are generated from the 655 projections with an evenly distributed angular spacing. The estimated dose reduction ratio is calculated based on the number of measured projection lines [16]. In both phantom studies, we use 219 projections and block 33% of illuminated area in each projection, resulting in the dose reduction ratio of around 75%. The proposed method is also compared with low-dose CBCT without scatter correction, where 163 projections are used, resulting in 75% dose reduction. Note that, the scatter estimation is also performed on the sparse projections.

For a quantitative error analysis, an additional set of projections is acquired with a fan-beam geometry, which narrows the collimator open width to around 10 mm on the detector for inherent scatter suppression. The resultant images are used as references. Image quality metrics are used to quantitatively evaluate the performance of the proposed method. For the selected ROIs, the CT number error is calculated as the square root of the mean square error (RMSE) and defined as

(2) $$\begin{array}{c} \text{RMSE}=\sqrt{\frac{1}{N}\overset{N}{\underset{i=1}{\sum }}{\left(\mu_{i}-\overset{-}{\mu_{i}}\right)}^{2}}, \end{array}$$

where *i* represents the index of ROI and *μ*_*i*_ is the mean reconstructed value inside the ROI and ${\overset{-}{\mu }}_{i}$ the value in the reference image and *N* is the total number of ROIs. The image contrast is calculated as

(3) $$\begin{array}{c} \text{contrast}=\left|\mu_{r}-\mu_{b}\right|, \end{array}$$

where *μ*_*r*_ is the mean reconstructed value inside the ROI and *μ*_*b*_ is the mean reconstructed value in the surrounding area.

## 3. Results

### 3.1. Catphan 600 Phantom Results

**Figure 2:**
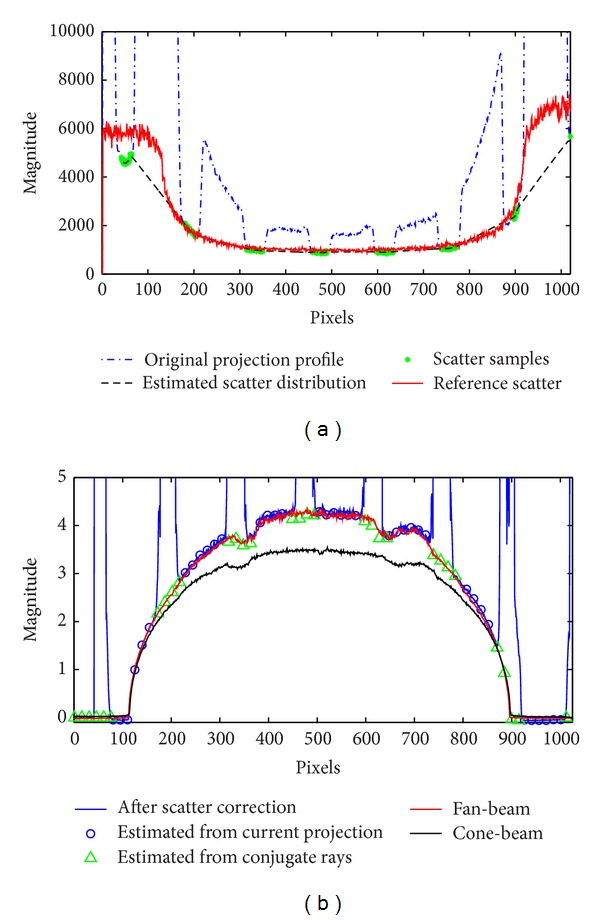
1D horizontal profiles of the scatter, projection, and line integral signals acquired from the Catphan 600 phantom: (a) estimated and reference scatter, original projection signals; (b) line integrals of CBCT projections with and without the proposed correction and with a fan-beam geometry. Different markers are plotted to demonstrate the data acquired from direct measurement (circle) and primary compensation (triangle).

Figure 2 shows the 1D horizontal profiles of scatter signals, raw projections, and line integrals of one projection on the Catphan 600 phantom. The reference scatter signals are obtained as the difference of the cone-beam and fan-beam projections. As seen in Figure 2(a), the estimated scatter profile using our method matches well with that of the measurement in the central region pixels (250–800) with an estimation error of less than 6.5%. Relatively large deviations are found around and outside the phantom boundary. However, the intensity of primary signals in these areas is high, which leads to a negligible estimation error of line integral.  Figure 2(b) shows the line integrals with and without the proposed method. The blue circles indicate the scatter corrected primary signals measured in the illuminated area. The missing primary signals due to the insertion of the beam blocker are compensated with their conjugate rays analytically and shown as the green triangles in Figure 2(b). Our method significantly enhances the intensities of the line integrals, which are close to those of the ground truth, that is, fan-beam CT.

**Figure 3:**
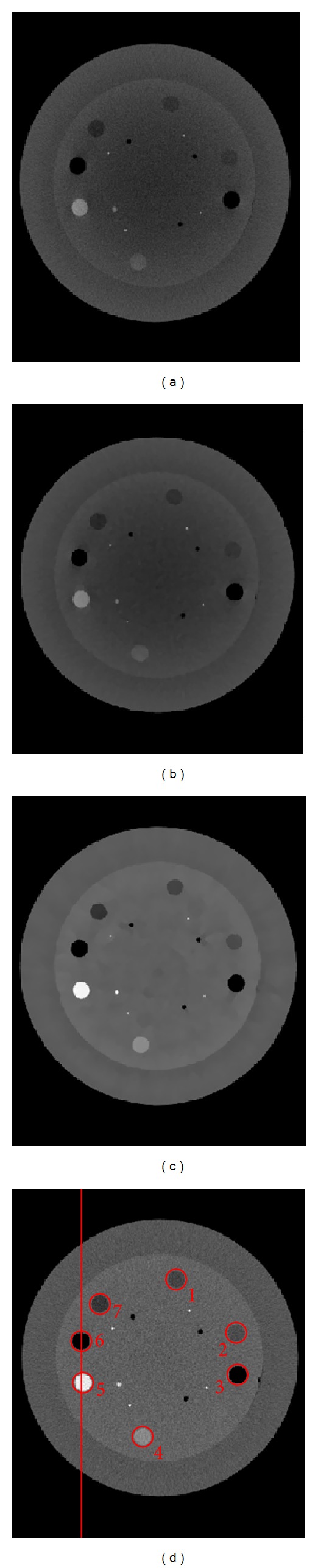
Axial views of the reconstructed Catphan 600 phantom. (a) CBCT without scatter correction using FDK algorithm and 655 projections; (b) low-dose cone-beam CT without scatter correction using**  **ABOCS and 163 projections (estimated 75% dose reduction); (c) CBCT using the proposed scatter correction and ABOCS reconstruction using 218 projections (estimated 75% dose reduction); (d) fan-beam CT as the ground truth using FDK reconstruction and 655 projections. The selected uniform ROIs are marked with red circles in (d). Display windows: [−400  600] HU.

**Figure 4:**
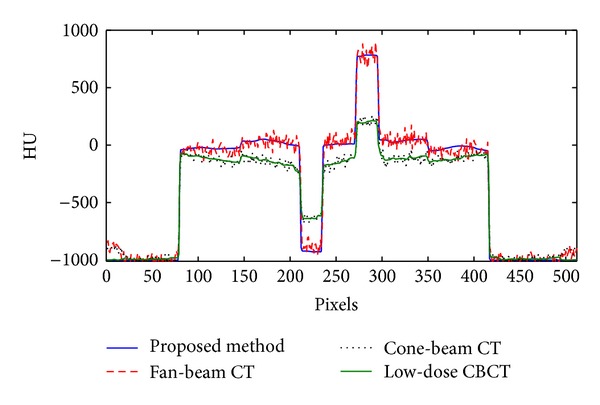
Comparison of 1D profiles of the CT images in Figure 3 taken along the straight line in Figure 3(d).

Figure 3 shows the reconstructed image with and without the proposed low-dose and scatter-free CBCT imaging scheme. Without scatter correction, the ABOCS reconstruction reduces the dose by 75%, however, severe shading artifacts are still observed (see Figures 3(b) and 4). Our proposed method significantly suppresses the shading artifacts (see Figure 3(c)). After improvement, the image quality is comparable to that of the reference (i.e., fan-beam CT in Figure 3(d)). For the quantitative evaluation of the performance using our method, the average CT numbers and contrasts are calculated for the contrast rods in one of the phantom slice. The results are summarized in Table 1 using those from the fan-beam CT as the references. In the selected ROIs, the proposed method reduces the mean CT number error from over 250 HU to around 24 HU and increases the contrast by a factor of 2.1**  **on the average.  Figure 4 shows the comparison of 1D profiles passing through two high contrast rods inside the phantom, as indicated by the red line in Figure 3(d).

### 3.2. Head Phantom Results

**Figure 5:**
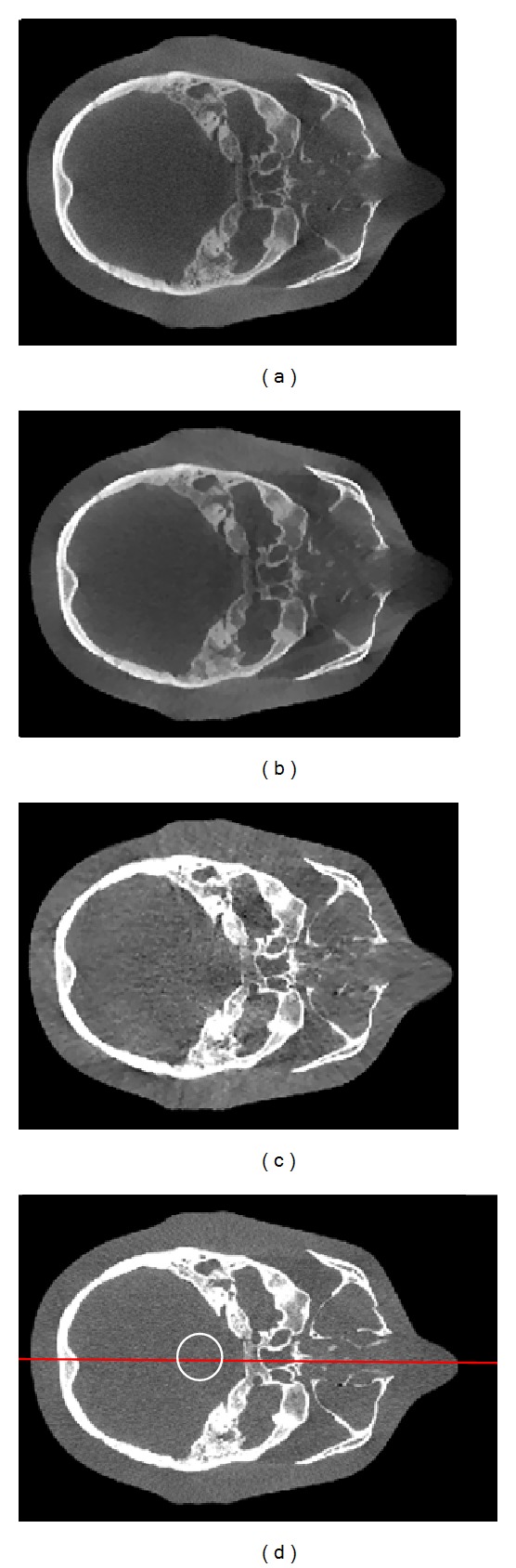
Axial views of the reconstructed head phantom. (a) CBCT without scatter correction using FDK algorithm and 655 projections; (b) low–dose cone-beam CT without scatter correction using**  **ABOCS algorithm and 163 projections (estimated 75% dose reduction); (c) CBCT using the proposed scatter correction and ABOCS reconstruction with 218 projections (estimated 75% dose reduction); (d) fan-beam CT using FDK reconstruction and 655 projections. In the central uniform area (marked with a white circle), the average CT numbers from (a) to (d) are −175, −180, 39, and 49 HU, respectively. Display windows: [−500  900] HU.

**Figure 6:**
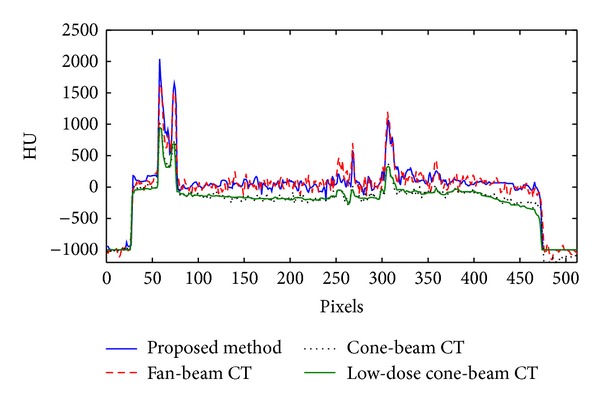
Comparison of 1D profiles along the central horizontal line as shown in Figure 5(d).

Figure 5 shows the axial views of the reconstructed head phantom images using the conventional FDK reconstruction, the low-dose ABOCS reconstruction, and the proposed method. The full-scan fan-beam CT image is generated as the ground truth. Similar to the Catphan 600 phantom results, the shading artifacts in Figures 5(a) and 5(b) are significantly suppressed with the proposed method. The mean CT number error is reduced from over 220 HU (Figures 5(a) and 5(b)) to 10 HU (Figure 5(c)) in the central uniform area as indicated by the white circle. The overall image uniformity of our result (Figure 5(c)) is close to that in the fan-beam result (Figure 5(d)), with only 25% radiation dose of a routine CBCT scan. The comparison of 1D profiles passing through the central horizontal line, as indicated by the red line in Figure 5(d), is shown in Figure 6.

## 4. Discussion

In this paper, we propose a low-dose and scatter-free CBCT imaging method in a single scan using a stationary beam blocker for scatter measurement and ABOCS reconstruction on the incomplete primary signals. Superior performance of the proposed method is demonstrated on the Capthan 600 phantom and an anthropomorphic head phantom.

For demonstration purposes, only 2D images are reconstructed in this work. In the future, we will extend our method to 3D reconstruction. Due to the huge size of system matrix, it is not practical to store the whole matrix in the computer memory for iterative CT reconstruction. Instead, we will formulate the multiplication of system matrix as a forward projection operation and speed up the calculation using hardware acceleration technique, for example, on a graphics processing unit (GPU) [12, 40].  The other issue with the 3D extension is that the cone angle can be as large as 6° the off-planes in the OBI system [41], which makes the small cone-angle approximation less accurate. Nevertheless, we would like to emphasize that the artifacts stemming from a large cone angle are generic issues in circular CBCT [42, 43]. For example, the small cone angle approximation is also used in 3D FDK reconstruction [34], the current standard algorithm implemented on commercial systems. For our applications of scatter correction with reduced projection measurement, we have shown in our previous study that the enlarged cone angle leads to negligible image quality degradation on clinical CBCT systems [17]. We would expect a similar performance of off-plane imaging for the proposed method in this paper.

## 5. Conclusion

In this paper, we propose a practical CBCT imaging method for dose reduction and scatter correction using a stationary blocker in a single scan. In the tabletop phantom studies, our method reduces the overall CT number error form 250 HU to less than 24 HU and increases the image contrast by a factor of 2.1 in the selected ROIs with only 25% dose of a conventional CBCT scan. Our method has the promise to become a practical solution for scatter correction and low-dose imaging on different CBCT systems.

## Figures and Tables

**Table 1:** Comparison of the averaged CT numbers and contrasts inside the contrast rods of the Catphan 600 phantom. The CT number errors are also shown in parentheses. The numbers of the ROIs are marked in Figure 3(d). All unites are in HU.

	ROI	1	2	3	4	5	6	7	RMSE
	Fan-beam CT	−131	−84	−895	248	776	−889	−211	
	CBCT with correction	−148	−107	−933	227	780	−921	−234	
CT number (HU)		(−17)	(−23)	(−38)	(−21)	(4)	(−32)	(−23)	24
	CBCT without correction	−239	−217	−646	−48	202	−639	−281	
		(−108)	(−133)	(249)	(−296)	(−574)	(250)	(−70)	250
	CT number improvement	91	110	211	275	570	218	47	

	CBCT with correction	169	118	934	201	753	933	253	
Contrast (HU)	CBCT without correction	88	55	452	112	322	456	126	
	Contrast improvement	1.93	2.13	2.07	1.79	2.34	2.05	2.02	

## References

[1] Brenner DJ, Hall EJ (2007). Computed tomography—an increasing source of radiation exposure. *New England Journal of Medicine*.

[2] Sykes JR, Amer A, Czajka J, Moore CJ (2005). A feasibility study for image guided radiotherapy using low dose, high speed, cone beam X-ray volumetric imaging. *Radiotherapy and Oncology*.

[3] Sorensen SP, Chow PE, Kriminski S, Medin PM, Solberg TD (2006). Image-guided radiotherapy using a mobile kilovoltage X-ray device. *Medical Dosimetry*.

[4] Murphy MJ, Balter J, Balter S (2007). The management of imaging dose during image-guided radiotherapy: report of the AAPM Task Group 75. *Medical Physics*.

[5] Kan MWK, Leung LHT, Wong W, Lam N (2008). Radiation dose from cone beam computed tomography for image-guided radiation therapy. *International Journal of Radiation Oncology Biology Physics*.

[6] Ding GX, Coffey CW (2009). Radiation dose from kilovoltage cone beam computed tomography in an image-guided radiotherapy procedure. *International Journal of Radiation Oncology Biology Physics*.

[7] Zou Y, Pan X, Sidky EY (2005). Image reconstruction in regions-of-interest from truncated projections in a reduced fan-beam scan. *Physics in Medicine and Biology*.

[8] Niu T, Zhu L (2012). Accelerated barrier optimization compressed sensing (ABOCS) reconstruction for cone-beam CT: phantom studies. *Medical Physics*.

[9] Tsaig Y, Donoho DL (2006). Extensions of compressed sensing. *Signal Processing*.

[10] Sidky EY, Pan X (2008). Image reconstruction in circular cone-beam computed tomography by constrained, total-variation minimization. *Physics in Medicine and Biology*.

[11] Sidky EY, Kao CM, Pan X (2006). Accurate image reconstruction from few-views and limited-angle data in divergent-beam CT. *Journal of X-Ray Science and Technology*.

[12] Jia X, Lou Y, Li R, Song WY, Jiang SB (2010). GPU-based fast cone beam CT reconstruction from undersampled and noisy projection data via total variation. *Medical Physics*.

[13] Tian Z, Jia X, Yuan K, Pan T, Jiang SB (2011). Low-dose CT reconstruction via edge-preserving total variation regularization. *Physics in Medicine and Biology*.

[14] Zhu L, Wang J, Xing L (2009). Noise suppression in scatter correction for cone-beam CT. *Medical Physics*.

[15] Zhu L, Xie Y, Wang J, Xing L (2009). Scatter correction for cone-beam CT in radiation therapy. *Medical Physics*.

[16] Dong X, Jia X, Niu T, Zhu L Low-dose and scatter-free cone-beam CT imaging: a preliminary study.

[17] Niu T, Zhu L (2011). Scatter correction for full-fan volumetric CT using a stationary beam blocker in a single full scan. *Medical Physics*.

[18] Endo M, Tsunoo T, Nakamori N, Yoshida K (2001). Effect of scattered radiation on image noise in cone beam CT. *Medical Physics*.

[19] Siewerdsen JH, Moseley DJ, Bakhtiar B, Richard S, Jaffray DA (2004). The influence of antiscatter grids on soft-tissue detectability in cone-beam computed tomography with flat-panel detectors. *Medical Physics*.

[20] Siewerdsen JH, Jaffray DA (2000). Optimization of X-ray imaging geometry (with specific application to flat-panel cone-beam computed tomography). *Medical Physics*.

[21] Boone JM, Seibert JA (1988). An analytical model of the scattered radiation distribution in diagnostic radiology. *Medical Physics*.

[22] Gao H, Fahrig R, Bennett NR, Sun M, Star-Lack J, Zhu L (2010). Scatter correction method for X-ray CT using primary modulation: phantom studies. *Medical Physics*.

[23] Gao H, Zhu L, Fahrig R (2010). Modulator design for X-ray scatter correction using primary modulation: material selection. *Medical Physics*.

[24] Zhu L, Bennett NR, Fahrig R (2006). Scatter correction method for X-ray CT using primary modulation: theory and preliminary results. *IEEE Transactions on Medical Imaging*.

[25] Maltz JS, Blanz WE, Hristov D, Bani-Hashemi A Cone beam X-ray scatter removal via image frequency modulation and filtering.

[26] Colijn AP, Beekman FJ (2004). Accelerated simulation of cone beam X-Ray scatter projections. *IEEE Transactions on Medical Imaging*.

[27] Kyriakou Y, Riedel T, Kalender WA (2006). Combining deterministic and Monte Carlo calculations for fast estimation of scatter intensities in CT. *Physics in Medicine and Biology*.

[28] Niu T, Zhu L (2010). Overview of X-ray scatter in Cone-beam computed tomography and its correction methods. *Current Medical Imaging Reviews*.

[29] Maltz JS, Gangadharan B, Vidal M (2008). Focused beam-stop array for the measurement of scatter in megavoltage portal and cone beam CT imaging. *Medical Physics*.

[30] Ning R, Tang X, Conover D (2004). X-ray scatter correction algorithm for cone beam CT imaging. *Medical Physics*.

[31] Wagner FC, Macovski A, Nishimura DG (1988). Dual-energy X-ray projection imaging: two sampling schemes for the correction of scattered radiation. *Medical Physics*.

[32] Kyriakou Y, Meyer M, Kalender WA (2008). Technical note: comparing coherent and incoherent scatter effects for cone-beam CT. *Physics in Medicine and Biology*.

[33] Niu T, Sun M, Star-Lack J, Gao H, Fan Q, Zhu L (2010). Shading correction for on-board cone-beam CT in radiation therapy using planning MDCT images. *Medical Physics*.

[34] Feldkamp IA, Davis LC, Kress JW (1984). Practical cone-beam algorithm. *Journal of the Optical Society of America A*.

[35] Zhu L, Strobel N, Fahrig R X-ray scatter correction for cone-beam CT using moving blocker array.

[36] Cho S, Lee T, Min J, Chung H (2012). Feasibility study on many-view under-sampling technique for low-dose computed tomography. *Optical Engineering*.

[37] Pua R, Min J, Yoo B, Kim KW, Cho G, Cho S Backprojection-filtration image reconstruction from partial cone-beam data for scatter correction.

[38] Dong X, Niu T, Jia X, Zhu L (2012). Relationship between X-ray illumination field size and flat field intensity and its impacts on X-ray imaging. *Medical Physics*.

[39] Wang J, Mao W, Solberg T (2010). Scatter correction for cone-beam computed tomography using moving blocker strips: a preliminary study. *Medical Physics*.

[40] Jia X, Yan H, Cervino L, Folkerts M, Jiang SB (2012). A GPU tool for efficient, accurate, and realistic simulation of cone beam CT projections. *Medical Physics*.

[41] Siewerdsen JH, Jaffray DA (2001). Cone-beam computed tomography with a flat-panel imager: magnitude and effects of X-ray scatter. *Medical Physics*.

[42] Zhu L, Yoon S, Fahrig R (2007). A short-scan reconstruction for cone-beam CT using shift-invariant FBP and equal weighting. *Medical Physics*.

[43] Zhu L, Starman J, Fahrig R (2008). An efficient estimation method for reducing the axial intensity drop in circular cone-beam CT. *International Journal of Biomedical Imaging*.

